# The effect of technology-based service characteristics on relationship quality in metaverse exercise services: a questionnaire survey of bicycle simulator users in Korea

**DOI:** 10.1186/s40359-024-01967-4

**Published:** 2024-09-02

**Authors:** Dong-Kyu Kim, So-Eun Lee, Sung-Un Park, Chulhwan Choi

**Affiliations:** 1 Department of Sport, Leisure, & Recreation, Soonchunhyang University, Asan-si, 31538 Republic of Korea; 2 College of Wesley Creative Convergence, Hyupsung University, Hwaseong-si, 18330 Republic of Korea; 3 Department of Sports Science, Hwasung Medi-Science University, Hwaseong-si, 18274 Republic of Korea; 4 Department of Physical Education, Gachon University, Seongnam-si, 13120 Republic of Korea

**Keywords:** Technology-based self-service, Metaverse exercise, Relationship quality

## Abstract

**Background:**

As non-face-to-face contact has become a daily routine owing to the development of science and technology and impact of the coronavirus disease (COVID-19) pandemic, online technology-based services (TBSSs) have been expanding. Consequently, in virtual metaverse exercise spaces, the number of participants using TBSS is increasing. This study aimed to examine the effects of TBSS characteristics on the quality of the relationship between service providers and users of metaverse exercise services.

**Methods:**

The participants were metaverse exercise service users in Korea, who were selected through purposive sampling (*n* = 254, 194 men, 61 women). A questionnaire survey was conducted to measure the participants’ TBSS characteristics (enjoyment, stability, usefulness, ease of use, and reliability) and relationship quality (satisfaction, trust, and commitment). We analyzed the frequency, validity, reliability, and descriptive statistics of the collected data. Moreover, correlation and multivariate multiple regression analyses were conducted.

**Results:**

Enjoyment, stability, usefulness, and reliability of TBSS had positive effects on satisfaction; however, ease of use did not have a significant effect. In addition, enjoyment, usefulness, ease of use, and reliability of TBSS had positive effects on trust; however, stability did not have a significant effect. Furthermore, enjoyment and reliability of TBSS had positive effects on commitment; however, usefulness, ease of use, and stability had no significant effects.

**Conclusions:**

This study provides metaverse exercise service providers with management strategies for attracting and retaining members. The results of this study could help develop effective TBSS and aid metaverse service companies facing a fiercely competitive environment.

## Introduction

As non-face-to-face contact has become routine due to the development of science and technology and the impact of the coronavirus disease (COVID-19) pandemic, online technology-based self-services (TBSSs) that do not require face-to-face contact with employees are expanding [1]. TBSS is defined as “any technological access means that allows customers to directly use services on behalf of members of the company providing services to customers” [2]. In other words, humans receive services by repeatedly interacting with technology, which includes unmanned, automated, and non-face-to-face self-service, such as kiosks, robot serving, and online-based drive-thru. As such, providers can provide consistent services and also reduce labor costs [3], while users can increase their service satisfaction due to the standardized service provision [2].

Social distancing and movement restrictions implemented in various countries during the COVID-19 pandemic led to a decline in overall physical activity [4], which, in turn, negatively affected people’s physical, mental, and social health [5]. To reduce the threat of COVID-19 infection, services without environmental restrictions providing a metaverse space that crosses the border between the virtual and real worlds are growing rapidly. During the COVID-19 pandemic, existing offline services were naturally replaced by online ones [6]. In the sports field, existing offline services were expanded online [7].

As technology advances, online and offline metaverse services, such as Peloton (Peloton Interactive, Inc., New York, USA), are being developed. Peloton is a fitness video-streaming service that primarily sells bicycles and simulators [8]. In other words, Peloton is a representative bicycle metaverse exercise service where participants exercise in different offline spaces and share or compete against each other in the online metaverse space. In addition to Peloton, several similar exercise devices and programs, such as YAFIT (Yanadoo Corp., Seoul, Republic of Korea), have emerged in Korea. After the start of the COVID-19 pandemic, metaverse exercise services have rapidly changed people’s daily lives. While the exercise and sports industry was a labor-intensive industry in the past, it is now technology-intensive and is launching new services based on various technologies.

Considering this social and environmental background, companies that provide TBSS, such as metaverse services, must pay attention to their relationship quality with customers according to the characteristics of the services they offer. In particular, companies must establish strategies to provide effective services to users and understand the impact of TBSS characteristics on the relationship quality with service beneficiaries (i.e., users) to gain a competitive advantage in the market. TBSS can be described based on five characteristics: enjoyment, stability, usefulness, ease of use, and reliability [7]. Users produce and receive services independently at service contact points without the assistance of employees [2]. TBSS focuses on providing services through interactions with technology in non-face-to-face environments [2, 9, 10]. TBSS changes the way customers, companies, and employees interact in a variety of fields and provides infinite possibilities for redesigning services [11].

Relationship quality refers to the relationship between the service provider and beneficiary and consists mainly of trust, commitment, and satisfaction [12]. These factors are considered key variables in the service field and have a significant impact on user behavioral intentions, such as re-registration and recommendation intentions [13, 14]. Research to improve relationship quality with customers in the service market has long been consistently conducted, and its importance has been increasing as competition in the market has grown. According to Rudolph et al. [15], companies should not only attract new members but also improve the relationship quality with existing members to gain a competitive advantage in the market. If the focus is on attracting new members in the early stages of service, retaining existing members is an important factor during the growth period, when the business expands. As metaverse exercise services are expanding rapidly, member retention strategies based on relationship quality are important [7].

The public’s interest in healthy living and exercise participation has been increasing since the COVID-19 pandemic; consequently, the market for new TBSS-based exercise services is expected to expand rapidly in the future. Service providers have a unique opportunity to differentiate themselves and increase user engagement and satisfaction by understanding and implementing the key TBSS characteristics that foster strong relationship quality between service providers and users [16]. By fostering a deeper connection with users through enhanced services, companies can build a loyal customer base, reduce churn rates, and achieve long-term business success [17]. This study examined the impact of TBSS characteristics of metaverse exercise services on the relationship quality between service providers and users. The results can provide meaningful information for developing effective TBSS, which could aid metaverse service companies facing a fiercely competitive environment.

### Metaverse exercise services

Metaverse exercise services refer to fitness and exercise platforms that operate in virtual reality (VR) or augmented reality (AR) environments [18]. These services leverage immersive technology to create interactive and engaging workout experiences that can be accessed remotely. Unlike traditional fitness applications, metaverse exercise services provide a holistic and interactive approach by simulating real-world exercise environments and incorporating elements of social interaction and gamification [19].

Metaverse exercise services represent a significant advancement in the field of fitness and wellness, combining the benefits of digital technology with the motivational aspects of social interaction and gamification [20]. The success of these services hinges not only on their ability to deliver a seamless, engaging, and effective exercise experience that meets the diverse needs of users but also on the technology-based self-service characteristics implemented by service providers and quality of the relationship between providers and users [20].

Therefore, the adoption and success of metaverse exercise services are closely tied to how well service providers leverage technology to create an intuitive, beneficial, and socially supportive environment [21]. Ensuring high relationship quality between service providers and users is crucial, as it fosters trust, satisfaction, and loyalty, which are essential for the long-term sustainability and effectiveness of these platforms [22].

### Human–technology interaction in metaverse exercise services

The interaction between humans and technology in metaverse exercise services is influenced by several key factors, including the aftermath of the COVID-19 pandemic, increased public interest in exercise participation, and advancements in the technological environment [23]. These factors provide a robust theoretical foundation for understanding how human–technology interaction impacts relationship quality between service providers and users.

The COVID-19 pandemic significantly altered lifestyle behaviors and heightened the focus on health and well-being. According to the Health Belief Model, individuals are likely to engage in health-related behaviors when they perceive a high threat to their health and believe in the effectiveness of the recommended health actions [24]. During the COVID-19 pandemic, while traditional fitness centers were closed or restricted, virtual exercise platforms became a necessary alternative, thereby driving users towards metaverse exercise services [25]. This increased reliance on digital platforms underscores the importance of effective human–technology interaction to ensure user engagement and satisfaction.

The surge in public interest in exercise participation can be contextualized through the theory of planned behavior (TPB) [26]. The TPB posits that intention, attitude, subjective norms, and perceived behavioral control influence an individual’s engagement in a behavior [27]. The COVID-19 pandemic has shifted societal norms and attitudes towards highly valuing physical fitness, increasing people’s intentions to engage in regular exercise. Metaverse exercise services cater to this heightened interest by providing accessible, flexible, and engaging exercise options [23]. Effective human–technology interactions in these services, such as real-time feedback, social connectivity, and gamified experiences, enhance users’ perceived behavioral control and positive attitudes, leading to higher participation rates and improved relationship quality [28].

Advancements in technology, particularly VR, AR, and artificial intelligence (AI), have revolutionized the delivery of exercise services [29]. The technology acceptance model (TAM) provides a framework to understand this phenomenon, suggesting that perceived ease of use and perceived usefulness are critical factors in technology adoption [25]. Metaverse exercise services leverage VR and AR to create immersive workout environments and AI to provide personalized training programs and real-time adjustments [30]. These technological enhancements improve the user experience by making the services intuitive and effective, thereby strengthening the relationship quality between users and service providers [31].

By leveraging these theoretical insights, metaverse exercise service providers can enhance user engagement, satisfaction, and loyalty, thereby achieving a competitive edge in the rapidly evolving market.

### The characteristics of technology-based self-service

TBSS provides an interface in which customers produce and perform services themselves, rather than receiving them directly from corporate organizational members [2]. Consumers directly contact information devices, such as kiosks, and receive services independently in a mutual relationship with technology [32]. Services were previously provided at points of contact with customers; therefore, the role of the service provider was important [33]. However, in a TBSS environment, the service characteristics perceived by customers during their interaction with technology have a significant impact on consumer behavior. Indeed, the recent proliferation of kiosk adoption has further increased the interaction between consumers and information devices [32]. The interaction between consumers and information devices is increasing [34, 35]. Therefore, consumers’ information accessibility has improved, significantly impacting their purchasing behaviors [36–38].

TBSS characteristics were first identified by Parasuraman [39], who suggested the concept of “technology readiness” and the importance of technology. In the previous service environment, five constructs (SERVEQUAL) were used: tangibility, reliability, responsiveness, assurance, and empathy [40]. However, in a TBSS environment, self-service takes place at the point of contact with the technology; therefore, the configuration concept must be adjusted to consider this [41]. According to Van Riel et al. [42], the usage attributes of TBSS are responsiveness, efficiency, and perceived credibility.

In particular, as TBSS characteristics vary depending on the characteristics of the customer, TBSS should be designed based on these characteristics [43]. Metaverse exercise services assist users work out efficiently and pleasurably by leveraging characteristics such as immersion, real-time interaction, accessibility, and personalized service [44]. As companies offer new services through TBSS, they must prioritize the quality of customer relationships based on the characteristics of the services provided. This is expected to enhance service efficiency, including reduced cost, increased convenience, and a greater provision of personalized service.

This study adopted the characteristics of metaverse exercise services presented in the existing literature and examined the enjoyment, stability, usefulness, ease of use, and reliability of metaverse exercise services perceived by users [7]. To explain each of them, (1) enjoyment means the positive emotions users feel when using technology-based self-service systems. (2) stability means the system operates consistently and reliably without unexpected disruptions. (3) usefulness means the system effectively helps users achieve their goals. (4) ease of use means the system is simple for users to learn and operate. (5) reliability means the system consistently performs as expected, building user trust [7, 45, 46].

### Relationship quality

Relationship quality refers to the depth of mutual relationships and consists of three factors: satisfaction, trust, and commitment [12, 47]. Satisfaction is a subjective evaluation of the differences between expectations prior to purchase and actual performance after purchase [48]. Customers form affective (emotional) responses after cognitive processing (expectation congruence and expectation disconfirmation) [49]. In other words, satisfaction can be defined as the cognitive state that a customer experiences in response to payment [50]. Mutual trust reduces uncertainty regarding service guarantees and the future [51]. In service relationships, guaranteeing services that share the risk of uncertainty increases trust [52, 53]. In relational situations, customers who trust service providers are likely to develop long-term relationships [54]. Commitment refers to an implicit or explicit promise to continue a relationship, in which the current relationship with another person is important, and is a major factor in successful relationships [55]. Greater perceived benefits of a specific company or brand by a consumer are associated with a higher degree of immersion [56, 57].

Recently, with the emergence of new services driven by technological advancements such as the metaverse [18], it is significant to re-evaluate the associations between metaverse service providers and users. To gain a competitive edge in the market, companies must enhance the quality of relationships with existing members in addition to attracting new ones [15]. In technology-based self-service environments, the service characteristics that the customers perceive during their interaction with technology significantly influences their behavior [58].

**Fig. 1:**
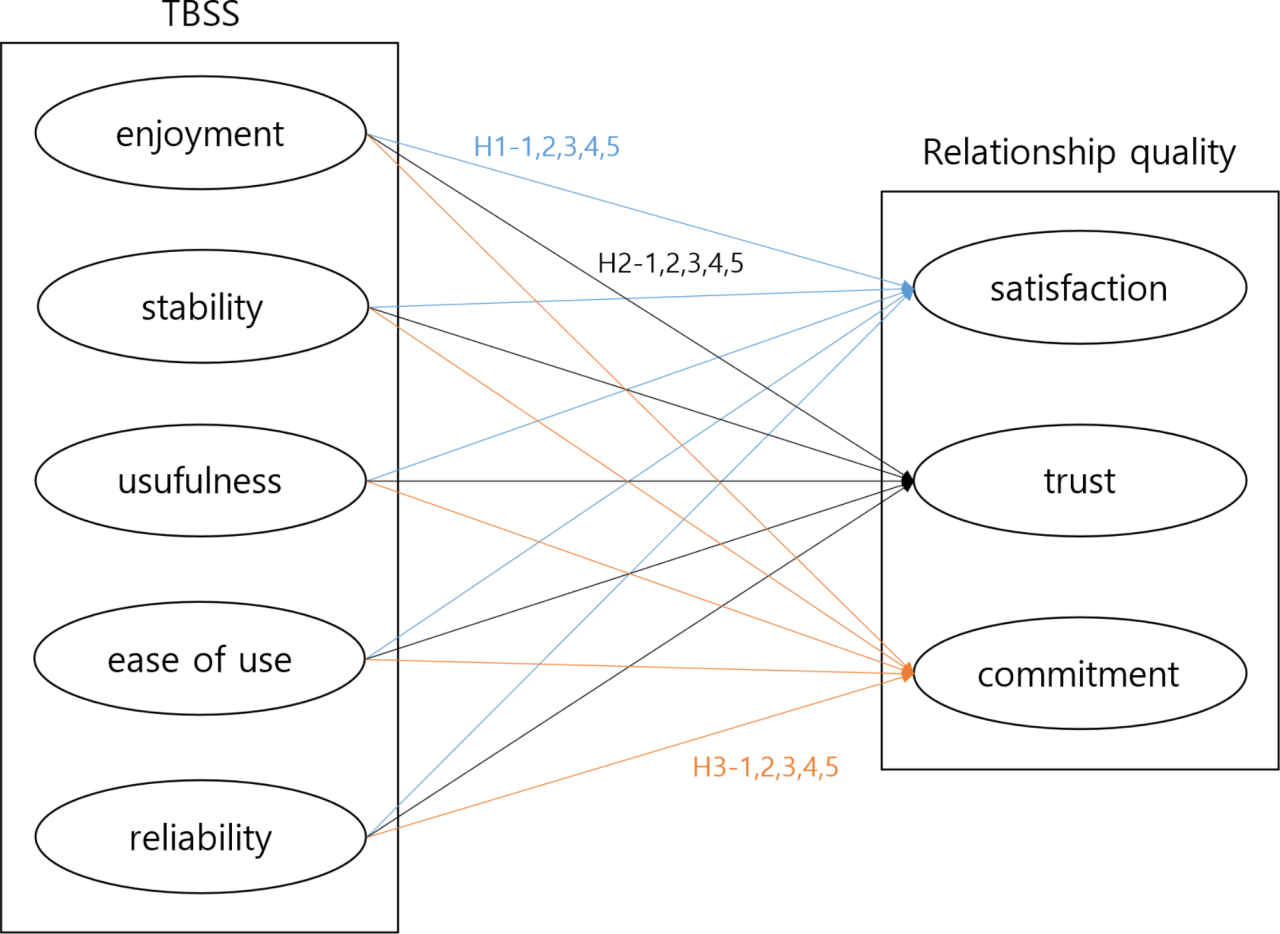
Research model

Hence, it can be inferred that a significant relationship exists between TBSS and the quality of relationships. Therefore, we established a research model (Fig. 1) and hypotheses to identify the relationships between the five characteristics of TBSS and three relationship quality constructs.

**H1-1**,** 1–2**,** 1–3**,** 1–4**,** 1–5.** The TBSS characteristics (enjoyment, stability, usefulness, ease of use, reliability) of the metaverse exercise service have a positive effect on satisfaction.

**H2-1**,** 2–2**,** 2–3**,** 2–4**,** 2–5.** The TBSS characteristics (enjoyment, stability, usefulness, ease of use, reliability) of the metaverse exercise service have a positive influence on trust.

**H3-1**,** 3 − 2**,** 3–3**,** 3–4**,** 3–5.** The TBSS characteristics (enjoyment, stability, usefulness, ease of use, reliability) of the metaverse exercise service have a positive effect on commitment.

## Methods

### Participants and procedures

**Fig. 2:**
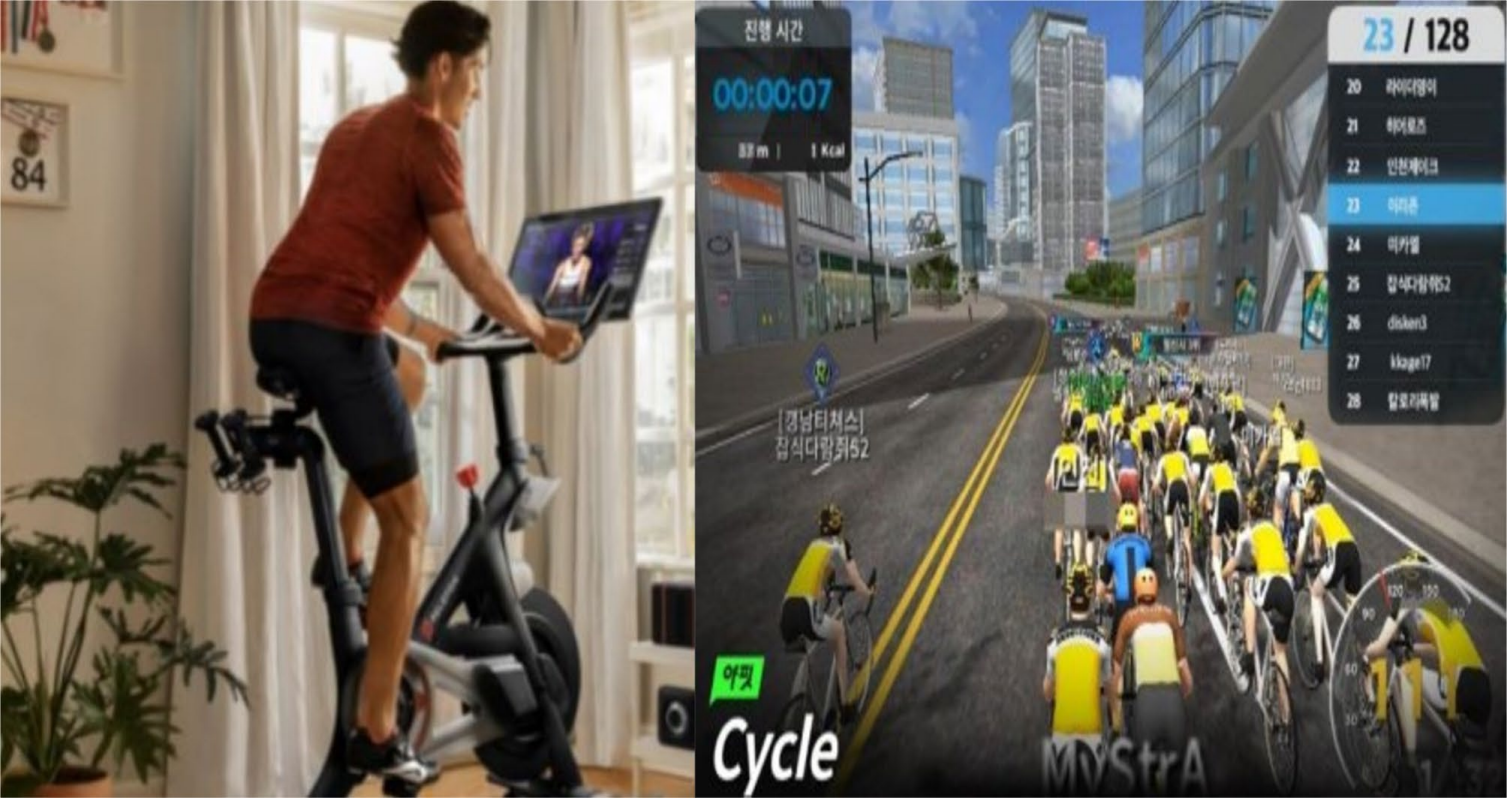
Peloton and YAFIT’s metaverse exercise services

This study conducted a questionnaire survey with individuals who had experience using the metaverse exercise service (Peloton and YAFIT). YAFIT is a bicycle simulator developed and used in the Republic of Korea [59]. YAFIT’s metaverse exercise services provided by Peloton and YAFIT are shown in Fig. 2.

Participants were selected using purposive sampling, a type of non-probability sampling. Specifically, we selected individuals aged 18–65 who currently participate in, or have previously participated in, metaverse workout services. However, we excluded students enrolled at the researcher’s university. The online survey was administered via Google Forms, and a link to the questionnaire was distributed to members of metaverse exercise services such as Peloton and YAFIT. The participants were informed about the purpose of the study and the voluntary nature of their participation. The general characteristics of the participants are listed in Table 1.

**Table 1:** General characteristics of the study participants

Variable	Category	*n*	Percentage (%)
Gender	Male	193	76.0
	Female	61	24.0
Age group	19–29 years old	151	59.4
	30–39 years old	38	15.0
	40–49 years old	57	22.4
	50–65 years old	8	3.1
Period of using metaverse exercise service	Less than 3 months	191	75.2
	3 months–less than 6 months	16	6.3
	6 months–less than 1 year	23	9.1
	More than 1 year	24	9.4
Perceived interest	High	48	18.9
	Medium	146	57.5
	Low	60	23.6
Total		254	100.0

Among the 254 valid questionnaires, there were 193 men (76%) and 61 women (24%); 151 participants (59.4%) were between 19 and 29 years old, 38 (15%) were between 30 and 39 years old, 57 (22.4%) were between 40 and 49 years old, and 8 (3.1%) were between 51 and 65 years old. Further, 191 people (75.2%) used the service for less than 3 months, 16 (6.3%) used the service for 3 to 6 months, 23 (9.1%) used the service for 6 months to 1 year, 24 (9.4%) used the service for over 1 year. Finally, 48 participants (18.9%) showed high perceived interest in the metaverse exercise service, 146 (57.5%) showed medium perceived interest in the metaverse exercise service, and 60 (23.6%) showed low perceived interest in the metaverse exercise service.

### Measures

The questionnaire comprised 27 questions to measure the eight observed variables: five TBSS characteristics (enjoyment, stability, usefulness, ease of use, and reliability) and three relationship quality characteristics (satisfaction, trust, and commitment). Responses were rated on a five-point Likert scale.

The TBSS characteristics were measured using 15 items (three items for each subcategory) employed by Seiders et al. [60]. Relationship quality was measured using 13 items employed by Kim and Choi [13]. In addition, four items regarding the general characteristics of the participants (gender, age, period of use of the metaverse exercise service, and perceived interest) were included.

To ensure the validity of the questionnaire used in this study, content validity was verified by experts in relevant fields. Subsequently, concentrated validity and discriminant validity were established through confirmatory factor analysis. The results of confirmatory factor analysis indicated that the fit index of the measurement model met the criteria (CFI > 0.90, IFI > 0.90 TLI > 0.90, RMSEA < 0.10) of the structural model suggested by Kline [61], as demonstrated in Table 2, confirming the acceptability of the analysis results.

Construct reliability (CR) and average variance extracted (AVE) values were calculated to analyze the centralized validity of each variable used in the study. The CR of all observed variables used in this study ranged from 0.831 to 0.952, while AVE ranged from 0.623 to 0.832. These values met the criteria of 0.7 or higher for CR and 0.5 or higher for AVE, as suggested by Anderson and Gerbing [62] and Fornell and Larcker [63], thereby confirming the centralized validity of all variables. Additionally, the AVE of the constituent concepts was compared with the squared correlation coefficient between these concepts to verify the discriminant validity; the AVE was found to be higher than the squared correlation coefficient, thus confirming the discriminant validity between the constituent concepts. The reliability analysis of the eight constituent concepts used in this study revealed that all variables exceeded the standard value of 0.70 recommended by Nunnally & Bernstein [64], ensuring the reliability of the items for each factor.

**Table 2:** Results of the confirmatory factor analysis

Factors	Items					β		SE	t		CR	AVE	α
Enjoyment	I enjoy participating in the metaverse movement.					0.910		-			0.936	0.829	0.929
	I find participating in the metaverse movement interesting.					0.956		0.041	25.664				
	I think participating in the metaverse movement is a new challenge.					0.844		0.047	19.563				
Stability	I am not concerned about performing incorrect movements while participating in the metaverse movement.					0.837		-			0.899	0.748	0.896
	I have no fear of difficulty in operating the device or injury while participating in the metaverse movement.					0.907		0.062	17.247				
	I have low concerns about safety accidents that may occur in the process of joining the metaverse movement.					0.840		0.064	15.877				
Usefulness	I believe that my overall athletic performance has improved through the metaverse exercises.					0.791		-	-		0.862	0.676	0.846
	I believe that smooth exercise performance is possible through metaverse exercises.					0.836		0.065	14.510				
	I think the metaverse movement is effective for many movement participants.					0.803		0.065	13.800				
Ease of use	I can easily participate in the metaverse movement.					0.765		-	-		0.875	0.700	0.855
	I think participating in the metaverse movement is easy.					0.889		0.080	14.420				
	I think participating in the metaverse movement is overall easy.					0.807		0.083	13.144				
Reliability	I believe that accurate movement performance is possible through metaverse exercises.					0.853		-	-		0.831	0.623	0.825
	I believe that the metaverse movement always provides consistent services.					0.777		0.059	14.331				
	I believe that it will work without problems even if unexpected situations arise during the metaverse exercise process.					0.730		0.069	13.119				
Satisfaction	I am overall satisfied with the metaverse exercise service.					0.922		-	-		0.952	0.832	0.937
	I think it was a good idea to use the metaverse exercise service.					0.921		0.040	25.245				
	The metaverse exercise service meets my expectations.					0.869		0.045	21.539				
	The metaverse exercise service provides effective exercise performance.					0.850		0.046	20.430				
Trust	I trust the metaverse movement service provider.					0.880		-	-		0.948	0.819	0.925
	I trust the services of the metaverse exercise service provider.					0.913		0.045	21.539				
	Metaverse movement service providers treat us with trust.					0.841		0.049	18.152				
	Metaverse exercise service providers are genuine.					0.852		0.048	18.637				
Commitment	I would be happy to make an additional effort to make a deal with a metaverse exercise service provider.					0.892		-	-		0.916	0.686	0.894
	I feel royalty from the metaverse exercise service provider.					0.740		0.053	14.429				
	I will maintain the relationship even if the performance of the metaverse exercise service provider is low.					0.719		0.059	13.783				
	I think of the relationship with the metaverse exercise service provider as a long-term partnership.					0.802		0.049	16.506				
	I have a desire to maintain a relationship with a metaverse exercise service provider.					0.821		0.050	17.229				
Model fit	X^2^	df	*p*	NFI	IFI		TLI			CFI		RMSEA	
	827.797	322	0.000	0.876	0.921		0.906			0.920		0.079	

### Statistical analysis

Frequency, validity, reliability, correlation, and multivariate multiple regression analyses of the collected data were conducted using SPSS 23.0 and AMOS 23.0. All statistical verification processes were conducted at a significance level of *p* < .05.

## Results

### Normality verification, descriptive statistics, and correlation analysis

The descriptive statistical analysis of the eight constructs indicated that the values ​​were within the range of ± 2 for skewness and ± 7 for kurtosis, confirming the normality of the data [65]. In addition, the correlation coefficients between all variables were lower than 0.85, indicating no multicollinearity concerns [61]. Table 3 presents the descriptive statistics and results of correlation analyses.

**Table 3:** Descriptive statistics and correlation coefficients

	Enjoyment	Stability	Usefulness	Ease of use	Reliability	Satisfaction	Trust	Commitment
1	AVE = 0.829							
2	0.258^**^	AVE = 0.748						
3	0.603^**^	0.470^**^	AVE = 0.667					
4	0.403^**^	0.496^**^	0.672^**^	AVE = 0.703				
5	0.423^**^	0.535^**^	0.697^**^	673^**^	AVE = 0.622			
6	0.631^**^	0.445^**^	0.713^**^	0.507^**^	0.624^**^	AVE = 0.832		
7	0.580^**^	0.479^**^	0.703^**^	0.639^**^	0.686^**^	0.784^**^	AVE = 0.819	
8	0.571^**^	0.345^**^	0.587^**^	0.507^**^	0.664^**^	0.709^**^	0.729^**^	AVE = 0.687
M	3.59	3.35	3.71	3.72	3.37	3.65	3.65	3.48
SE	0.902	0.900	0.837	0.832	0.856	0.808	0.760	0.750
Skewness	− 0.582	0.006	− 0.463	− 0.371	0.044	− 0.330	0.083	0.083
Kurtosis	0.459	− 0.502	0.478	0.025	− 0.058	0.241	− 0.118	0.672

** p < .01.

### Hypothesis testing

A multivariate multiple regression analysis was conducted to test the study hypotheses, and the results are presented in Table 4.

**Table 4:** Results of the multivariate multiple regression analysis

Variables		B	t	*p*	np^2^	Power
Satisfaction	Intercept	0.547	3.151	0.002	0.039	0.881
	Enjoyment	0.284	6.398	0.000	0.142	1.000
	Stability	0.096	2.205	0.028	0.019	0.594
	Usefulness	0.347	5.346	0.000	0.103	1.000
	Ease of use	− 0.066	-1.161	0.247	0.005	0.212
	Reliability	0.215	3.694	0.000	0.052	0.957
Trust	Intercept	0.557	3.492	0.001	0.047	0.936
	Enjoyment	0.208	5.094	0.000	0.095	0.999
	Stability	0.072	1.811	0.071	0.013	0.438
	Usefulness	0.186	3.116	0.002	0.038	0.874
	Ease of use	0.159	3.026	0.003	0.036	0.894
	Reliability	0.246	4.584	0.000	0.078	0.995
Commitment	Intercept	0.890	5.102	0.000	0.095	0.999
	Enjoyment	0.280	6.260	0.000	0.136	1.000
	Stability	− 0.034	− 0.785	0.433	0.002	0.122
	Usefulness	0.031	0.480	0.632	0.001	0.077
	Ease of use	0.031	0.546	0.585	0.001	0.085
	Reliability	0.435	7.420	0.000	0.182	1.000

The results demonstrated that enjoyment, stability, usefulness, and reliability had positive effects on satisfaction. However, ease of use did not have a significant effect on satisfaction. TBSS characteristics explained 60.3% (R^2^ = 0.603) of the total variance in satisfaction.

Enjoyment, usefulness, ease of use, and reliability had positive effects on trust. However, stability did not significantly affect trust. TBSS characteristics explained 62.1% (R^2^ = 0.621) of the total variance in trust.

Enjoyment and reliability had positive effects on commitment. However, stability, usefulness, and ease of use did not have significant effects. TBSS characteristics explained 53.6% (R^2^ = 0.536) of the total variance in commitment.

## Discussion

The results revealed that TBSS characteristics and relationship quality were positively correlated. Metaverse exercise services involve repeated real-time interactions between service providers and users. This is due to a combination of factors, such as personalized experience, enhanced interaction, increased accessibility, improved user experience, real-time feedback, motivation, and social support. Therefore, TBSS characteristics are expected to provide users with positive experiences, which in turn improves relationship quality, leading to higher satisfaction and continued use of the service.

Our findings revealed that enjoyment, stability, usefulness, and reliability of the metaverse exercise service had positive effects on satisfaction. The metaverse exercise service, which takes place in a variety of technological environments [66], allows multiple people to log in simultaneously and participate together, performs for multiple electronic devices that transmit and receive data, provides an interesting challenge program to present participants’ exercise performance, and presents consistent programmed services. However, ease of use did not have a significant effect. Metaverse exercise services can be used anytime, anywhere [67]. The metaverse exercise service provides a touch-type intuitive interface. However, the normal level of expectations perceived by participants accustomed to digital services has already been formed [68]; thus, ease of use did not significantly affect satisfaction. Therefore, metaverse service providers should enhance enjoyment, stability, usefulness, and reliability to increase user satisfaction and loyalty.

The metaverse environment repeatedly interacts with various technologies. As users participate in the service, user satisfaction directly impacts their intention to continue using the service. In particular, as the YAFIT bicycle simulator is an online self-service, it requires continuous development of interesting content in the interaction between humans and technology and effective exercise performance. This study indicated that usefulness and enjoyment had the strongest influence on satisfaction. In addition, operators need to establish differentiated strategies to attract new sports participants and convert general sports participants. Marketing strategies focusing on interest and stability are required to attract new participants, whereas promotional activities emphasizing usefulness and reliability are required for existing participants.

Furthermore, enjoyment, usefulness, ease of use, and reliability of the metaverse exercise service had a positive effect on trust. The enjoyment of participating in a new metaverse exercise service, its usefulness as a complement to existing exercise, the convenience of participating easily anytime and anywhere, and consistent service quality have a positive effect on trust in service providers [59]. However, stability did not have a significant effect. The metaverse exercise service is performed with multiple people in a virtual space; however, in the offline space, this exercise is performed alone through self-interaction with technology [69]. This increases the risk of incorrect movement performance, safety accidents, and injury. This relates to the failure to provide problem-solving technologies for stability. This study found that the average value of stability was the lowest. Moreover, the participants generally used the metaverse exercise service for a short time. Therefore, service providers should expand content to enhance enjoyment, usefulness, ease of use, and reliability to increase the trust among participants, thereby improving customer relationships and increasing retention rates.

To date, the metaverse market has strived to create environments as similar to reality as possible. While maintaining this, providers should consider including arcade-type (surreal) content in the future. Services should differentiate between realistic and arcade versions and emphasize the enjoyment and usefulness of arcade versions in providing a type of catharsis that cannot be experienced in reality. This requires creating strategies that communicate with each other to ensure that the service provider can be trusted, allowing users to provide feedback on operations, and providing online and offline linked services through offline invitation events.

Furthermore, enjoyment and reliability positively affected commitment. The metaverse exercise service connects reality to a 3D virtual space. Even if users participate in exercises in a limited space in reality, they can experience new services in which they compete with various people in a three-dimensional metaverse space through online technology [70]. The challenging process of competing among participants in a virtual exercise space that cannot be experienced in reality [71] has a positive effect on commitment. In addition, in this process, trust in the service has a positive effect on commitment, as people exercise according to a consistently programmed service and compete in the same environment. However, stability, usefulness, and ease of use did not significantly affect commitment. This is due to the graphics of virtual spaces, which are far from reality, and the relatively lower perceived usefulness and ease of exercising in a general environment. As such, metaverse exercise services continue to be perceived as a supplement rather than substitute for offline exercise. Providers should improve the enjoyment and reliability of their services to foster stronger user commitment, which could help increase user loyalty and retention.

Commitment to service providers has been identified as an antecedent factor that significantly influences loyalty, such as through regular partnerships [72]. Metaverse spaces are virtual three-dimensional spaces, and awkward virtual graphics that differ from reality can interfere with user commitment. Frequent changes in the service environment, such as usage rules, systems, and costs, can also hinder user commitment. Therefore, users should be alert. In addition, providers should strengthen contents that allow repeated interactions between them and users to increase user immersion. This could help promote metaverse exercise services as a substitute rather than a complement to offline exercise.

Thus, by identifying the relationship between TBSS characteristics and relationship quality, this study provides theoretical and practical insights for enhancing the growth of the metaverse exercise service market. However, this study focuses on improving service efficiency through TBSS, thus it is significant to validate the use of the TAM model in subsequent studies as TAM can predict behavior by explaining the process of users’ acceptance of new technologies [73]. Hence, a study that combines TBSS and TAM theory by analyzing service acceptance and actual usage experience in a multidimensional way may be vital for formulating more useful customer-centered marketing strategies. Moreover, integrating TAM into future research will assist in identifying key factors such as perceived ease of use and usefulness, which are critical for understanding user adoption and satisfaction in metaverse environments [73]. This combined approach can provide a more comprehensive framework for developing effective engagement strategies and enhancing user experiences in the metaverse exercise service market.

This study had several limitations. First, our sample included mostly men. Therefore, follow-up studies should generalize the results for other samples. Second, as the quality of relationships is a complex effect of various factors, additional variables must be introduced in follow-up studies. Furthermore, future studies should present three-dimensional results based on analyses of differences between groups. Third, this study collected data in the early stages of metaverse exercise services in Korea. Despite concerted efforts to achieve a balanced sample distribution, 75.2% of the participants reported using the metaverse service for less than three months. This highlights an important aspect of the current metaverse exercise market, namely, the large proportion of recent adopters due to this being a new field. Fourth, selection of this study’s subjects and data collection proved challenging as the initial research was conducted on the metaverse exercise service project. Therefore, multivariate multiple regression analysis was conducted as opposed to structural equation modeling (SEM) as it can yield valid results even with a relatively small sample size. In future, studies can focus on verifying the complex relationship between the variables by employing SEM. Consequently, our findings primarily reflect the experiences and perceptions of these new users. Future research should aim to include participants with a wider range of usage durations to ensure a more comprehensive understanding of the long-term effects and relationship quality between service providers and users. Moreover, as related industries grow and change rapidly, step-by-step research in the early, middle, and late stages is required.

## Conclusions

This study examined the impact of TBSS characteristics on the quality of relationships between service providers and users of metaverse movement services. This study offers valuable insights into the factors affecting metaverse exercise services. Our results suggest that providers should improve the TBSS characteristics of metaverse exercise services to increase user satisfaction, trust, and commitment, thereby fostering strong customer relationships and providing companies with a competitive advantage in the burgeoning metaverse exercise market. Our findings are particularly important because public interest in healthy living is growing, and exercise participation is increasing.

Our findings highlight the importance of personalized experiences, seamless interaction, and immersive environments in the metaverse. Service providers can use these insights to create tailored and compelling exercise services that not only meet but exceed user expectations. Moreover, our findings provide a strategic roadmap for service providers to innovate and thrive in a competitive landscape, ultimately contributing to the overall advancement of TBSS in the exercise industry.

## Data Availability

No datasets were generated or analysed during the current study.
